# Decontamination-Induced Modification of Bioactivity in Essential Oil-Based Plasma Polymer Coatings

**DOI:** 10.3390/molecules26237133

**Published:** 2021-11-25

**Authors:** Olha Bazaka, Karthika Prasad, Igor Levchenko, Mohan V. Jacob, Kateryna Bazaka, Peter Kingshott, Russell J. Crawford, Elena P. Ivanova

**Affiliations:** 1 School of Science, STEM College, RMIT University, Melbourne, VIC 3000, Australia; s3729979@student.rmit.edu.au (O.B.); russell.crawford@rmit.edu.au (R.J.C.); 2 School of Engineering, The Australian National University, Canberra, ACT 2601, Australia; Karthika.Prasad@anu.edu.au (K.P.); katia.bazaka@anu.edu.au (K.B.); 3 Plasma Sources and Applications Centre, NIE, Nanyang Technological University, Singapore 637616, Singapore; levchenko.igor@nie.edu.sg; 4 College of Science & Engineering, James Cook University, Townsville, QLD 4810, Australia; mohan.jacob@jcu.edu.au; 5 Department of Chemistry and Biotechnology, Swinburne University of Technology, Hawthorn, VIC 3122, Australia; pkingshott@swin.edu.au; 6 Australian Research Council (ARC) Industrial Transformation Training Centre in Surface Engineering for Advanced Materials (SEAM), Swinburne University of Technology, Hawthorn, VIC 3122, Australia

**Keywords:** plasma polymer, atmospheric pressure plasma, antibacterial polymer coatings

## Abstract

Plasma polymer coatings fabricated from *Melaleuca alternifolia* essential oil and its derivatives have been previously shown to reduce the extent of microbial adhesion on titanium, polymers, and other implantable materials used in dentistry. Previous studies have shown these coatings to maintain their performance under standard operating conditions; however, when used in e.g., a dental implant, these coatings may inadvertently become subject to in situ cleaning treatments, such as those using an atmospheric pressure plasma jet, a promising tool for the effective in situ removal of biofilms from tissues and implant surfaces. Here, we investigated the effect of such an exposure on the antimicrobial performance of the *Melaleuca alternifolia* polymer coating. It was found that direct exposure of the polymer coating surface to the jet for periods less than 60 s was sufficient to induce changes in its surface chemistry and topography, affecting its ability to retard subsequent microbial attachment. The exact effect of the jet exposure depended on the chemistry of the polymer coating, the length of plasma treatment, cell type, and incubation conditions. The change in the antimicrobial activity for polymer coatings fabricated at powers of 20–30 W was not statistically significant due to their limited baseline bioactivity. Interestingly, the bioactivity of polymer coatings fabricated at 10 and 15 W against *Staphylococcus aureus* cells was temporarily improved after the treatment, which could be attributed to the generation of loosely attached bioactive fragments on the treated surface, resulting in an increase in the dose of the bioactive agents being eluted by the surface. Attachment and proliferation of *Pseudomonas aeruginosa* cells and mixed cultures were less affected by changes in the bioactivity profile of the surface. The sensitivity of the cells to the change imparted by the jet treatment was also found to be dependent on their origin culture, with mature biofilm-derived *P. aeruginosa* bacterial cells showing a greater ability to colonize the surface when compared to its planktonic broth-grown counterpart. The presence of plasma-generated reactive oxygen and nitrogen species in the culture media was also found to enhance the bioactivity of polymer coatings fabricated at power levels of 10 and 15 W, due to a synergistic effect arising from simultaneous exposure of cells to reactive oxygen and nitrogen species (RONS) and eluted bioactive fragments. These results suggest that it is important to consider the possible implications of inadvertent changes in the properties and performance of plasma polymer coatings as a result of exposure to in situ decontamination, to both prevent suboptimal performance and to exploit possible synergies that may arise for some polymer coating-surface treatment combinations.

## 1. Introduction

When it comes to controlling biofilm growth on surfaces, plasma-based modification of surfaces is often a popular choice to prevent the initial stages of bacterial attachment from taking place [1,2,3,4]. This occurs because partially ionised plasmas have the chemistry of a species that is both highly reactive and often unique [5,6], and this allows one to drive reactions that would not otherwise happen under equivalent thermochemical conditions [7,8,9]. In practice, this means that highly uniform polymer coatings can be deposited on surfaces at lower temperatures and without the need for solvents and catalysts [10,11]. These coatings can prevent the formation of biofilms by allowing the surface to elute active biocides, as in the case of plasma polymer coatings from essential oils [12,13], or through contact killing, as seen in coatings comprised of networks of sharp carbon nanotubes or walls [14,15,16].

Increasingly, plasmas are also becoming a popular tool for the in situ decontamination of biomaterials, exhibiting activity against a wide range of Gram-negative and Gram-positive bacteria, including those present in thick biofilms, as well as against other pathogenic microorganisms. Since the direct removal of cells and biofilms from surfaces relies on their exposure to significant quantities of RONS, UV, mild heating and air drying [17,18], it is important to consider the possibility of the modification of the biomaterial surface as an inadvertent consequence of such a treatment, and subsequent changes that are likely to occur in the interactions of these modified surfaces with fluids, proteins and cells.

The aim of this study was to investigate how a brief exposure to therapeutic doses of atmospheric pressure argon plasma may change the bioactivity and antibacterial performance of biocide-eluting polymer coatings fabricated using plasma polymerization [19]. As the delivery of plasma-generated species to bacterial targets typically takes place across a layer of fluid that encapsulates surfaces in vivo, materials may become exposed to chemically reactive species that are generated and remain in the plasma-treated liquid for a considerable period of time. These long life species include hydroxyl radicals (OH·), hydrogen peroxide (H_2_O_2_), ozone (O_3_), superoxide (O_2_^−^), nitric oxide (NO·), and various derivatives, e.g., peroxynitrite (OONO^−^ and ONOOH) that form from interactions of these species with molecules in gas and liquid phases [20]. At the same time, exposure to high flows of warm gas of the plasma jet would result in the rapid drying of the surfaces.

Hence, this study considered both the direct treatment of clean and dry samples with plasma, the effect of introducing plasma-generated species into the media used to culture cells on these biomaterials, and the combination of the two treatments. Furthermore, to understand whether inadvertent changes in the surface chemistry and morphology and the subsequent cell-surface interactions would take place in other plasma-synthesized carbon-based antimicrobial materials, non-eluting plasma polymer coatings grown from the same precursor were also examined. Unlike eluting plasma polymer coatings, materials fabricated at higher input power levels lose the inherent activity of the precursor due to precursor fragmentation and increased levels of cross-linking.

## 2. Results and Discussion

### 2.1. Surface Properties and Chemical Composition of As-deposited Plasma Polymer Coatings

**Figure 1:**
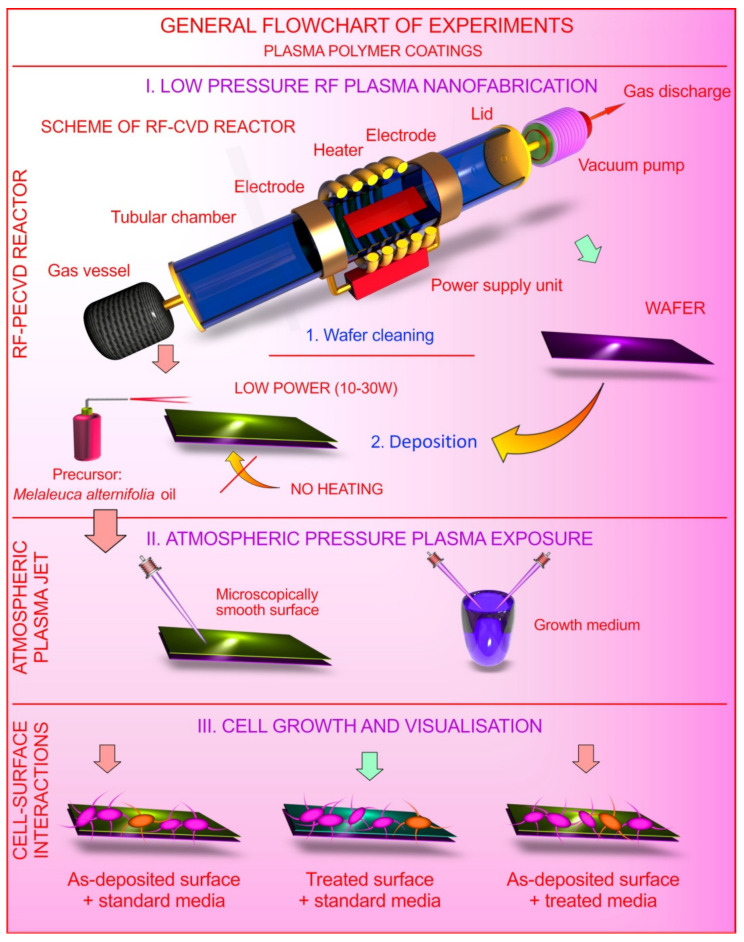
Schematic depiction of the experimental process. (**i**) plasma polymers are fabricated from the vapors of the *Melaleuca alternifolia* essential oil that deposit as a thin solid coating over virtually any substrata, in this case polyethylene terephthalate (PET), at 10–30 W input radio frequency (RF) power (for mechanism of polymerization please refer to [19]); (**ii**) a subset of these samples was exposed to atmospheric pressure argon plasma for up to 60 s, and their properties characterized; separately, aqueous media were activated using a similar atmospheric pressure argon plasma treatment; (**iii**) cells were seeded onto the surfaces of treated and untreated (control) polymer coatings, and their attachment and viability were characterized; cells were also grown on the surfaces of control polymer coatings in the presence of plasma-activated culture media.

Optically transparent (*k*→0 over the 500–1000 nm wavelength range) plasma polymer coatings were deposited on the surfaces of polyethylene terephthalate (PET) substrata (Figure 1) and their thickness estimated using a Cauchy model, as described in [21]. Plasma polymer coatings with a thickness of ~500 nm were selected because previous studies had found that films of lower thickness deposited on the surface of lotus-like titanium substrata may possess a reduced ability to retard bacterial attachment and subsequent biofilm formation [12]. This is likely because the antibacterial coatings based on essential oils, and synthesized using RF plasma polymerization, have a complex mechanism of activity that combines prevention of attachment due to a favorable combination of surface chemistry and surface topography, as well as killing of attached microorganisms through elution of biologically active molecules into the near-surface region [22,23]. In the case of thinner films, their ability to elute sufficiently high doses of antimicrobial agents may be lower, leading to less effective retardation of microbial proliferation and biofilm growth.

**Figure 2:**
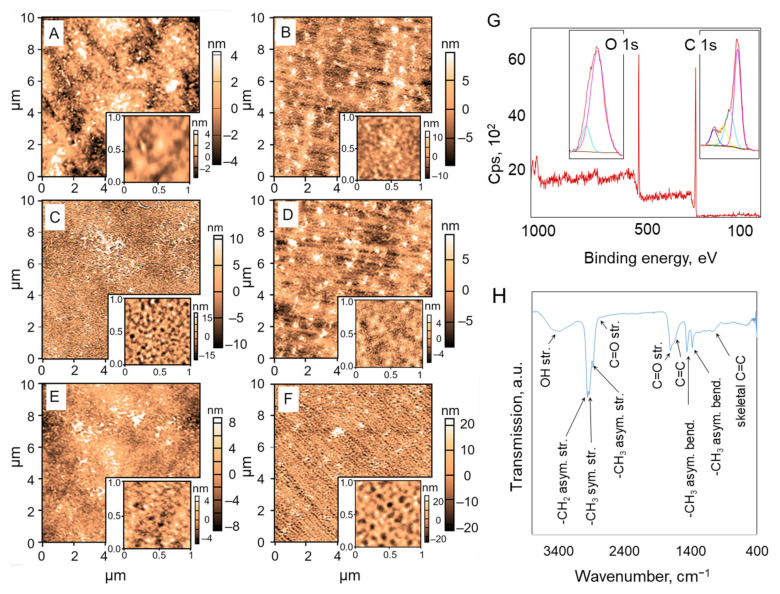
Surface morphology and chemical composition of plasma polymer coatings with a thickness of 500 nm. (**A**–**F**) Representative atomic force microscope (AFM) micrographs of surfaces of coatings deposited on PET substrata (**A**) at 10 W (**B**), 15 W (**C**), 20 W (**D**), 25 W (**E**), and 30 W (**F**). Scanning area for the main image is 10 × 10 µm, scanning area for the insets is 1 × 1 µm. (**G**) X-Ray Photoelectron Spectroscopy (XPS) wide spectrum for coatings deposited at 15 W. The inset shows the high-resolution scans for oxygen and carbon. (**H**) Fourier Transform Infrared (FTIR) spectrum for coatings deposited at 15 W.

AFM examination revealed substrate surfaces with a significantly different roughness profile (Figure 2A–F). As the input energy increased, the root mean squared (RMS) roughness *R*_q_ was first found to increase from 3.2 nm to 6.0 nm for samples fabricated at 10 W and 15 W, then reduced to 2.2 nm (20 W) and 1.7 nm (25 W), and once again began to increase for polymer coatings fabricated at 30 W (Table 1). The maximum peak height *R*_max_ followed a similar trend, changing from 22.5 nm (10 W) to 7.6 nm (25 W) to 33.8 nm (30 W). It is possible that at lower RF power levels (10–15 W), the level of monomer fragmentation was low, and the deposition was a combination of chemical polymerisation-type reactions in the gas phase, with surface chemical reactions being initiated by mild ion bombardment. Further reactions could then be taking place with plasma-activated monomer species and their fragments, with the subsequent physical adsorption of neutral monomer species and their oligomers and their entrapment within the framework of the growing coating matrix.

Once the power was increased to 20–25 W, monomer fragmentation in the gas phase became more efficient, with smaller molecular fragments being delivered to the surface. More intense ion bombardment of the surface promotes chemical reactions at the surface, resulting in a more homogenous, smoother film that is dominated by cross-linked branched hydrocarbons. The subsequent increase in the RF power to 30 W further intensified the extent of ion bombardment, leading to the removal of polymer coating material from the surface via etching and desorption of fragments due to the higher temperature of the substrate. The combination of etching and desorption may be responsible for the increased roughness of the resultant surface, with a significantly larger depth of valleys (at 33.8 nm for 30 W, as opposed to 18.2 nm obtained using a power level of 15 W) supporting this possibility. Indeed, the maximum depth of pits and valleys (*R*_min_*)* for samples fabricated at 30 W was found to be more similar to that of samples subjected to pure Ar plasma, where surface etching was found to be the dominant process.

The XPS results presented in Figure 2G and Table 1 confirm that as the RF power was increased, the dissociation of oil constituents, many of which contain alcohol groups, became more pronounced, leading to their transformation into hydrocarbon-rich polymer chains, and a gradual reduction in the oxygen content in the polymer matrix. The slight increase in the oxygen content for the coatings deposited at 30 W was most likely due to the post-synthesis interactions of the polymer coating with the ambient air. The location of the carbon and oxygen peaks that dominate the atomic composition of the coatings was found to be similar regardless of the deposition power, yet their magnitude differed, suggesting the formation of an increasingly hydrocarbon-rich material. Deconvolution of the peaks suggested that oxygen is present in the two binding states as C--O/C=O/Si—O (at ~530 eV) and *O--C=O (at ~531 eV), whereas carbon is in C-C/C-H, C-O, C=O and O-C=O (at 285, and ~286.5, 288 and 289 eV, respectively).

This was confirmed by analysing the FTIR spectra of the coatings (representative spectrum shown in Figure 2H). The N1s peak that appeared in some of the spectra was fitted with two peaks at ~400.0 and 402 eV, corresponding to chemical states where nitrogen atoms are attached to carbon atoms in the polymer coating change in the form of either amine or amide, and where nitrogen atoms are bonded to a single oxygen atom (C-N-O, N-C^+^) [22]. Where N1s peak lacked fine structure to assist fitting, the width of the peak suggested contributions from more than one chemical state of the element. Nitrogen is believed to originate from post-deposition ageing reactions of the unreacted monomer fragments on the surface of the freshly deposited polymer coating with ambient air [24].

For the deposition power range 10–30 W, the FTIR spectra show a similar shape, with difference in peak magnitude related to the reduction in the oxygen moieties (at ~3450 and 1708 cm^−1^). The relationship between the magnitude of peaks associated with the stretching and bending of CH_2_ and CH_3_ groups (e.g., 1450 and 1370 cm^−1^) suggested an increase in the cross-linking of carbon chains for coatings fabricated at higher RF powers, which has previously been linked to the reduced elution of active species for this type of coating [22,25,26] and, consequently, their reduced antibacterial activity [12,27].

### 2.2. Effect of Atmospheric Pressure Ar Plasma Treatment on Plasma Polymer Surfaces

**Figure 3:**
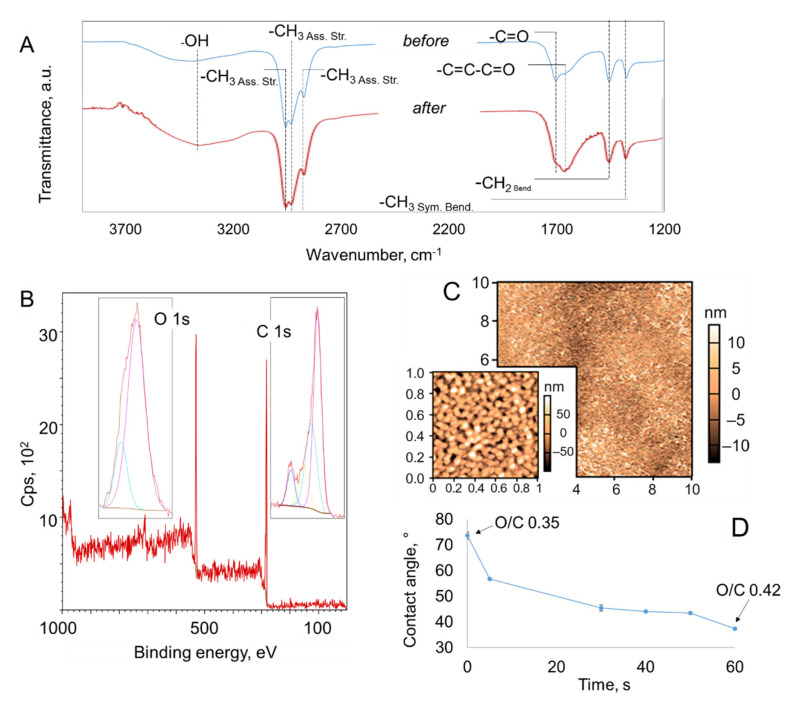
Effect of 60 s of direct exposure to Ar plasma on polymer coating fabricated at 15 W on PET. (**A**) FTIR spectra before and after treatment. (**B**) A typical XPS wide scan spectrum of treated polymer coating. Inset: high resolution spectra for oxygen and carbon. (**C**) AFM images after treatment. Scan size 10 × 10 µm. Inset scan size 1 × 1 µm. (**D**) Contact angle evolution as a function of treatment time. Oxygen to carbon ratio based on XPS atomic fractions shows an increase in oxygen content within the topmost layer of the polymer coating.

Exposure of the surfaces of polymer coatings to an Ar plasma jet resulted in a notable increase in the quantity of oxygen-bearing groups on the surface of the polymer coating. The FTIR spectra collected in the transmission mode enabled the probing of the bulk chemistry of the coating, whereas XPS was used to capture changes in the 10 nm-top-most surface layer of the coating. Examination of both spectra suggests that exposure of the substrate to the plasma jet resulted in a significant oxidation of the surface, with a significant quantity of oxygen atoms being added to the surface (Figure 3). The atomic fraction of oxygen on the surface increased for all samples. For coatings fabricated at 15 W, the oxygen increased from 26 to 30%. For other coatings, the oxygen fraction increased from 25.1 to 29.7% (for 10 W), from 22.9 to 25% (for 20 W), from 22.8 to 25.3% (for 25 W), and from 21.4 to 24.5% (for 30 W). It should be noted that even prior to the treatment, the polymer coating surfaces were rich in oxygen groups.

These additional oxygen atoms can come from several sources. First, during plasma polymerisation, oxygen moieties present in the monomer molecules and their fragments are retained within the polymer matrix. In the case of the deposition process used here, polymer coatings fabricated at lower RF power tended to have a greater fraction of O atoms in their structure.

Second, immediately after deposition, exposure of the coatings to the ambient air leads to a change in surface chemistry, with polymer coatings possessing a significant number of radicals on their surface being more susceptible to reactions with oxygen species in air. Through this mechanism, hydrocarbon-rich coatings deposited at higher RF powers gain a significant quantity of -OH groups on their surfaces in the form of low molecular weight oxidized materials. This has been demonstrated previously using XPS depth profiling, where etching is used to remove layers of polymer coating in-between XPS scans [28]. It should be noted that these low molecular weight oxidized materials can be easily removed by exposing the surface to a polar solvent, e.g., water, and thus cannot be relied upon as a reliable means of controlling cell-surface interactions.

Third, during argon plasma treatment, oxygen species that are produced as a result of interactions between Ar metastable species and ambient air have sufficiently long lives to allow them to reach and actively modify the surface, introducing -OH hydroxyl (the broad 3100–3600 cm^−1^ band on FTIR spectra, Figure 3A), –COOH carboxylic acid (~1640 cm^−1^), and C=O ketone (~1730 cm^−1^) groups. While the magnitude of these peaks changed as a result of argon plasma treatment, there were no new peaks introduced. This was consistent with the findings from the XPS spectra, where it was found that while the atomic fraction of oxygen increased, the position of the peaks that made up the O 1s and C 1s peaks remained in approximately the same position.

While air is rich in ambient nitrogen and nitrogen molecules, and these molecules would be excited as a result of interactions with the plasma-generated Ar metastable species and photons (as evident from the optical emission spectrum of Ar plasma in air), and then carried towards the surface by the jet, the very short lifetime of these species means that the excited and ionized nitrogen species are unlikely to reach and interact with the polymer coating surface to a significant extent. For example, for coatings fabricated at 15 W, the argon plasma treatment resulted in an increase in the atomic fraction of oxygen, from 26.0 to 29.6 %. Deconvolution of the O 1s spectrum showed oxygen was in two bonding states, as *O--C=O (at ~531 eV) and C--O/C=O (at ~530 eV). When compared to the unmodified plasma polymer coating, the relative fraction of *O--C=O was greater, whereas the fraction of C--O/C=O was lower, at ~20 vs. 13%, and 80 vs. 86.6, respectively (Figure 3, Table 2). Deconvolution of the C 1s spectrum showed four peaks, corresponding to C-C/C-H (at 285 eV), C-O (at ~286.5 eV), C=O (at ~288 eV) and O-C=O (at ~289 eV). The relative fraction of C-C/C-H was lower in the treated polymer coating when compared to its untreated counterpart, at 58 vs. 55%, respectively, whereas the relative fraction of states corresponding to carbon bonded to oxygen increased. It should be noted the noise in the spectra may conceal low levels of nitrogen that would be present on all plasma polymer coatings due to interactions with ambient air.

In addition to chemical modification, it is not unusual for the surfaces exposed to plasma-generated oxygen species and photons to experience material removal (*e.g.,* etching) and material redistribution across the surface. AFM examination of the surfaces revealed that while the RMS roughness of the samples increased, particularly when examining images with a scanning size of 1 µm × 1 µm, the increase was not significant. For example, for samples fabricated at 15 W (Table 2), the RMS roughness increased from ~6.0 to ~7.3 nm (for 1 µm × 1 µm) and from ~5.2 to 5.5 nm (10 µm × 10 µm). The maximum peak height also increased slightly for 1 µm × 1 µm scans, decreasing substantially from ~54.8 to ~34.3 nm when surfaces were visualised using the scanning size of 10 µm × 10 µm. It should be noted that just as water (or any other polar solvent) can be used to remove low molecular weight oxidized materials that remain on the surface after the coating is first exposed to the ambient air post-synthesis, these low molecular weight fragments can be displaced by exposing the surface to heat or the stream of the plasma jet. Thus, it is possible that the removal of these loose molecular fragments is responsible for the reduction in the maximum peak height at that scale. The maximum depth of pits and valleys (*R_min_*) did not change significantly, from ~18.2 nm to 22.0 nm (for 1 µm × 1 µm) and from ~20.3 nm to 18.7 nm (for 10 µm × 10 µm), confirming that the flow removal of loosely attached fragments and not necessarily etching was the primary mechanism behind the changes in surface morphology.

It is also possible that the initial removal of low molecular weight fragments is followed by the formation of a similar type of molecular fragments (as the polymer chains are attacked by the plasma-generated oxygen species). Nodule-like agglomerates of similar low molecular weight fragments have been shown to appear on the surfaces of polyethylene terephthalate, polyethylene and polypropylene treated with a similar atmospheric pressure plasma jet operated at 10 kV [29]. The plasma generated in this study is less intense and has a lower temperature, which may provide an explanation for the less pronounced surface restructuring observed in our study when compared to the aforementioned example.

Collectively, the changes in the chemistry and topography of the polymer coatings have led to a substantial increase in the hydrophilicity of coatings, with water contact angles changing from > 70° to 56.8° after 5 s of treatment, and to < 40° after 60 s of treatment. It should be noted that the decrease in the contact angle as a function of plasma treatment time is not linear, with the contact angle decreasing rapidly within the first 5–10 s, and then remaining fairly stable after 30 s of treatment. This observation is consistent with surface oxidation being the main mechanism responsible for plasma-surface interactions.

It is possible that substantially extending the treatment time would lead to a more significant change in the morphology and bulk structure of the polymer coating, due to gradual degradation of polymer chains via oxidation further promoted by UV irradiation and mild heating. Prolonged exposure of plasma polymers made of γ-terpinene (one of key constituents of tea tree oil) to UV–A (λ  =  350  ±  25 nm), UV–B (λ  =  300  ±  25 nm) and UV–C (λ  =  254  nm) light for 24, 48 and 672  h in ambient led to significant changes in the chemistry of the polymer, with UV-C inducing significant photochemical degradation and oxidation even under oxygen-poor conditions [30].

### 2.3. Effects of Atmospheric Pressure Ar Plasma Treatment on Bacterial Cell Growth on Polymer Coatings

#### 2.3.1. Effect of Substrate Treatment on Attachment of Bacterial Cells

Plasma polymers of tea tree oil retard the attachment and proliferation of bacterial cells through a combination of surface chemistry and morphology, and by killing cells that manage to attach through the localized elution of biocidal fragments. These eluted fragments then interfere with the bacterial cellular membrane, lodging themselves into the phospholipid bilayer to perturb the normal cellular structure and function, as well as transport across the membrane to form complexes with intracellular proteins through hydrogen bonds and hydrophobic interactions; and to induce intracellular reactive oxygen species (ROS) production that leads to DNA damage [31].

**Figure 4:**
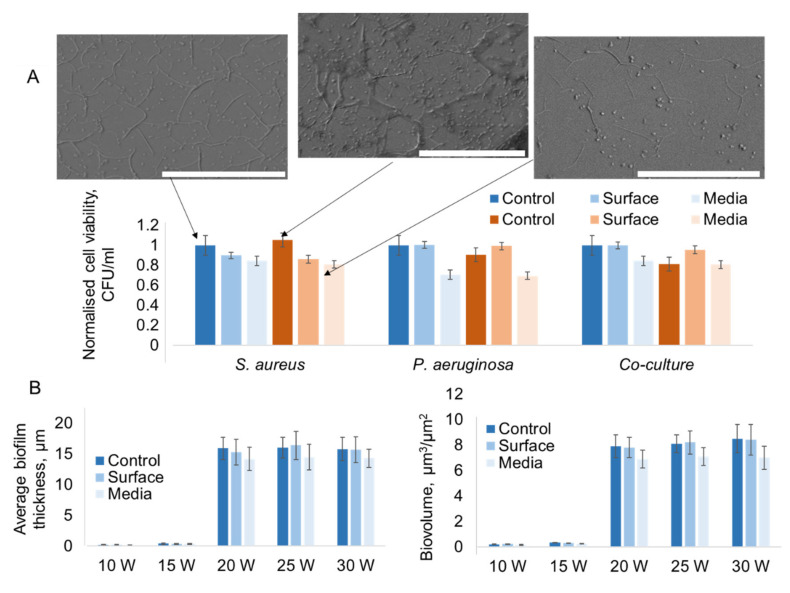
(**A**) Viability of *S. aureus*, *P. aeruginosa* bacterial cells and their mix culture when cultured on the surfaces of plasma polymer-modified PET substrata, normalised against colony forming units (CFU) for the “control biofilm” for each culture type. Inset: SEM images of *S. aureus* attachment after 24 h of incubation, scale bar 50 µm. Data shown for polymer coatings fabricated at 10 W, incubation time of 24 h. Blue bars: inoculum cells harvested from a mature biofilm (grown on the surface of culture plate for 72 h), red bars: from a planktonic culture. (**B**) Average biofilm thickness and biovolume of *P. aeruginosa* cells grown for 24 h from for polymer coatings fabricated at different RF power.

Treatment of surfaces of polymer coatings with argon plasma rendered these surfaces more hydrophilic without significantly changing their roughness and morphology. Figure 4A summarizes the viability of cells attached on 10 W polymer coating surfaces before and after argon plasma treatment. When inoculant cells were sourced from a mature biofilm, it was observed that the plasma treatment reduced the extent of attachment of the *S. aureus* cells, while the attachment and viability of *P. aeruginosa* and mixed cell populations remained similar. The likely explanation is that the treatment of polymer coatings resulted in the generation of loosely attached fragments rather than a change in the structure (and thus the elution profile) of the surface. These fragments are easily displaced from the surface, reverting the surface to a state that closely resembles that of the unmodified (as-deposited) polymer coating. Once removed, these fragments may provide an additional boost to the baseline antibacterial activity of the polymer coating, resulting in the lower number of *S. aureus* cells attaching to the surface. It is also possible that these fragments would have greater antibacterial activity against *S. aureus* cells when compared to *P. aeruginosa* cells.

When bacterial cells for inoculation were obtained from planktonic cultures, plasma treatment of the surface again enhanced the ability to retard attachment of *S. aureus* cells to the same extent as observed for the biofilm-derived cells. For *P. aeruginosa* cells, the colony forming units on the surfaces of plasma-treated polymer coating surfaces were found to be greater in number than that observed on the as-deposited polymer coating surface. This is possibly because the change in the contact angle and concomitant change in surface morphology would enable a greater extent of attachment by *P. aeruginosa* cells. *P. aeruginosa cells* are moderately hydrophilic, with a water contact angle of ∼43°, zeta potential of −14.4 ± 0.7 mV and mobility of −1.12 (μm/s)/(V/cm), whereas *S. aureus* cells are more hydrophobic, with a water contact angle of ∼72°, zeta potential of −35.2 ± 1.0 mV and mobility −2 (μm/s)/(V/cm) [23]. The plasma-treated surfaces on which mixed-species cells were grown also showed greater counts of viable cells, most likely attributable to the improved attachment and survival of *P. aeruginosa* cells. It should be noted that while the numbers of attached viable cells in the *P. aeruginosa* and mixed-species experiments recorded on plasma-treated surfaces was greater than that observed on the as-deposited samples, the values were similar to those obtained for biofilm-derived cells on plasma-treated surfaces.

For polymer coatings fabricated from tea tree oil at higher RF power, e.g., 20–30 W, their antibacterial activity was lower when compared to 10 W polymer coatings due to increased fragmentation during polymer coating synthesis that leads to a loss of the important chemical structures responsible for the antimicrobial activity of the oil and the coating. Figure 4B shows the biofilm thickness for *P. aeruginosa* grown on polymer coatings fabricated at different RF powers, where inoculum cells were derived from a mature biofilm. Figure 4B also shows the corresponding biovolume for these biofilms. Treatment of these polymer coatings with atmospheric pressure argon plasma reduced the contact angle of these coatings; however, this did not significantly improve the antimicrobial properties of these materials. This is because the treatment was short and mild, and as such did not induce any significant structural transformation or change in morphology that could have changed the mechanism by which the cells attached to these surfaces. Similarly, this treatment process did not change the bulk properties of the polymer coating, and thus the fragments that these polymer coatings elute.

#### 2.3.2. Effect of Plasma-activated Media on Attachment of Bacterial Cells

When the water in the cell culture medium was replaced with plasma-activated media during the cell incubation process, exposing the polymer coatings to plasma-generated species for the duration of incubation, the number of viable cells was reduced across all sample groups for both biofilm- and planktonic-derived cells, and mixed cultures (Figure 4A). *P. aeruginosa* cells appeared to be more sensitive to the synergistic action of polymer coatings and plasma-activated media when compared to that of the *S. aureus* cells. This is possibly due to greater susceptibility of *P. aeruginosa* cells to plasma-generated species when compared to Gram-positive cocci, which has been previously reported by Mai-Prochnow et al. [17]. Examination of the attachment patterns of *S. aureus* cells grown on polymer coating surfaces in the presence of plasma-activated media (Figure 4A inset) did not reveal any significant differences from cells attached onto as-deposited and plasma-modified polymer coatings grown in standard media.

The mechanism that may underpin this synergy is complex, as it is likely to involve both the interactions between plasma-generated RONS directly with cells, as well as with bioactive fragments eluted by the polymer coating. These fragments, particularly for polymer coatings deposited at 10–15 W, would include significant quantities of unmodified or minimally fragmented monomer species. These fragments, particularly oxygenated terpenes, may react with plasma-generated hydrogen peroxide, -OH and ozone, and other reactive species to produce a wide range of highly oxygenated biologically active derivatives with various degree of stability. The presence of metal ions may further catalyse these reactions, with photo-oxidation and auto-oxidation also taking place. The species produced in this way may not only be more biochemically active, but also target slightly different antimicrobial pathways. In parallel, plasma-generated RONS will directly target cells, specifically cell membrane integrity and permeability, interfering with intracellular redox balance, directly damaging protein and nucleic acid cellular components [20,32,33]. It has been suggested that these species may also change the gene activity and trait expression of the bacteria [34]. Damage to membrane integrity may in turn facilitate the transfer of polymer coating-generated antimicrobial agents into cells, where the simultaneous oxidative damage from RONS and plasma-generated species may prevent the cell from active repair and result in a more efficient killing of the microorganism.

It should also be noted that in these experiments, to investigate whether the presence of polymer film would interfere or enhance the antimicrobial activity of argon plasma treatment, plasma activated water was added to culture media immediately prior to its use for cell incubation on the surfaces of the polymer coating. While this approach does not perfectly mimic the processes that would take place during direct plasma treatment of cells residing on or in the proximity of polymer coating samples in vivo, it enables the decoupling of effects arising from plasma-generated chemical species from those arising from plasma-generated UV, heating or drying. Similarly, treatment of complete culture media containing salts, sugars and proteins produces a very complex chemistry that is heavily dependent on the chemistry of the media and introduces a multitude of organic peroxides (RO_2_) [32,35]. In the same way, UV and heating may promote certain chemical reactions not only in the media but also on the surface of the biomaterial. For example, photoexcitation of the carbon nanomaterial may promote these interactions, with photochemical formation of C_60_O from C_60_ reported via reaction of ^1^O_2_ with the lowest triplet excited state of C_60_ [36].

#### 2.3.3. Effect of Plasma Treatment on Attachment of Mammalian Cells

**Figure 5:**
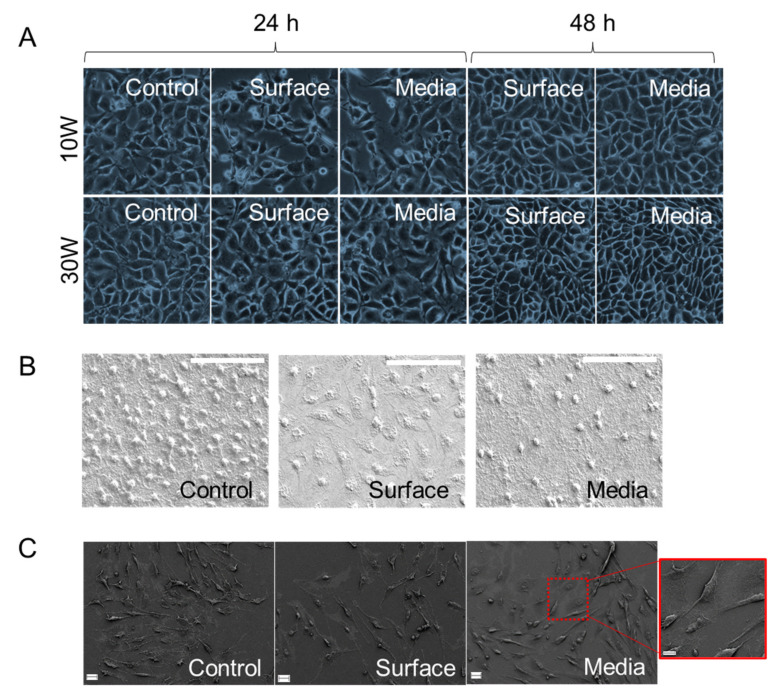
(**A**) Phase contrast images of wells containing fibroblasts grown in the presence of the coatings after 24 h and 48 h of incubation, scale bar 100 µm, and (**B**) SEM images of fibroblasts attached on the surfaces of substrata after 48 h of incubation, scale bar 50 µm. (**C**) SEM images of osteoblasts attached on the surfaces of substrata after 48 h of incubation, scale bar 20 µm, scale bar for inset 10 µm. For all images: “Control” denotes as-deposited surface and standard media, “Surface” is plasma treated surface and standard media, “Media” is as-deposited sample and plasma treated media. Data shown for polymer coatings fabricated at 10 W.

Plasma polymer coatings from tea tree oil derivatives have been previously shown to be biocompatible with all eukaryotic cell lines tested, independent on the RF used for the synthesis of the polymer coatings. This is because the quantities of antimicrobial agents eluted by these polymer coatings, even the most bioactive 10 W polymer coatings, are not sufficient to induce any notable toxicity in much larger eukaryotic cells. Figure 5A shows the growth of fibroblasts in the presence of as-synthesized and plasma-treated polymer coatings in the presence of standard or plasma-activated media.

Both plasma treatment and incubation in plasma-activated media appears to reduce the extent of cell attachment onto the 10 W polymer coatings, whereas the attachment tendencies on 30 W polymer coatings remain unaffected. The levels of plasma-generated radicals retained in the media are sufficiently high to induce damage in bacterial cells but too low to induce oxidative stress beyond the level that healthy cells could withstand. On the contrary, mild doses of plasma have been shown to promote cell growth and proliferation, and promote differentiation in MC3T3-E1 cells [37]. Hence, in the case of the plasma treatment of 10 W polymer coatings, the observed results could possibly be explained by the formation of loosely attached polymer fragments that can rapidly release larger than usual quantities of the active agents, making the surfaces less suitable for eukaryotic cell attachment. This can temporarily slow down the attachment and proliferation of cells on the surface. Furthermore, if these fragments are not released, there is a possibility for steric repulsion being responsible for the reduced cell-surface interactions, both for mammalian and bacterial cells [38]. Furthermore, it is also important to consider the effect of protein adsorption on the surface as a possible reason for reduced bioactivity of the surface. After 48 h, the attachment patterns were very similar between as-deposited and plasma-treated polymer coatings. Because 30 W polymer coatings do not have large quantities of bioactive agents within their structure, the impact from the loosely attached polymer fragments entering the cell culture environment is minimal, and cells can undergo attachment and proliferation in virtually the same way as they would on as-synthesised surfaces.

Incubation in plasma-activated media was associated with a similar temporary decline in fibroblast attachment for 10 W polymer coatings. While the ‘dose’ of plasma-generated species generated in the aqueous media during 60 s treatment was not on its own sufficient to induce any damage in cells, it may have been sufficient to slightly delay cell attachment when coupled with bioactive agents released by the 10 W polymer coatings. As mentioned previously, even in their as-deposited state, the surfaces of these polymer coatings are likely to contain on their surface a layer of loosely attached oxygenated material that is easily displaced when the polymer coating is exposed to a polar solvent. When coupled with reactive species in the plasma media, these fragments may be sufficient to temporarily inhibit fibroblast attachment. SEM images of attached fibroblasts show healthy well spread morphology of cells attached to all 10 W polymer coating surfaces, with unmodified polymer coating surfaces showing the highest number of attached cells (Figure 5B). Thus, while the attachment of these cells may be slowed down, their shape, size and ability to spread over the surface indicated minimal toxicity of the surfaces across all the sample groups. The attachment of osteoblasts showed a very similar pattern to fibroblasts (Figure 5C), with a slightly higher number of cells attached to as-deposited polymer coatings, and cells size, shape and morphology being similar across all sample groups. It should be noted that Figure 5 shows data for 10 W polymer coatings. Polymer coatings deposited at 15–30W showed very similar biocompatibility for both cell lines.

## 3. Materials and Methods

In this study, vapors of *Melaleuca alternifolia* oils, commonly known as tea tree oil, was used as a precursor for the synthesis of plasma polymerized antibacterial coatings. Oils were purchased from Australian Botanical Products and used without any additional processing.

### 3.1. Preparation and Characterisation of Polymer Coatings

The fabrication of polymer coatings was performed using a custom-made RF-PECVD reactor, with the RF energy (10–30 W) used to ignite and sustain the plasma delivered by the Navio RF generator (13.56 MHz, 1.2 kW max). Polyethylene terephthalate (PET) slides were used as substrata. The generator was capacitively coupled to the quartz tube using two external copper electrodes and an impedance matching network. The deposition was performed in the absence of external heating, with the temperature of the substrate never exceeding 30 °C over the duration of the deposition. The removal of contaminants from the surface prior to coating was achieved by first evacuating the chamber, flushing the tube with argon, and then exposing the surface to 1 min of argon plasma. The brevity of exposure coupled with the low RF energy ensured minimal etching-induced modification of substrata surface morphology. After cleaning the surface with plasma, the flow of argon was stopped and the plasma switched off, and the chamber was once again evacuated to the pressure of 50 mTorr. At this point, the vapours of *M. alternifolia* essential oil were released into the chamber at the flow rate of ~0.5 sccm. Plasma polymer coatings were deposited for 30 min at a pressure of 100–200 mTorr to achieve coatings with thickness of approximately 500 nm, as estimated from Ψ and Δ ellipsometric data collected at incidence angles φ = 55, 60, and 65° over the 200−1000 nm wavelength using a variable angle spectroscopic ellipsometer (model M-2000, J. A. Woollam Co., Inc., Lincoln, NE, USA). The optical transparency (*k*→0) of the coating over the 500−1000 nm region and its assumed macroscopic homogeneity enabled an estimation of the thickness and roughness of the coating using a Cauchy model [22]. The bulk and surface chemistry of the coating were characterised using Perkin Elmer Spectrum 100 Fourier transform infrared (FTIR) spectrometer (Perkinelmer Inc., Boston, MA, USA) and Kratos-Axis XPS (Shimadzu, Kyoto, Japan) equipped with a monochromatic Al Kα X-ray source (hν = 1486.6 eV) operating at 150 W. Binding energies for Au 4f_7/2_, Ag 3d_5/2_, and Cu 2p_3/2_ at *E*_b_ of 84.0, 368.3, and 932.7 eV, respectively, were used to calibrate the instrument’s energy scale. Bombardment with low-energy electrons were used to offset surface charging in the polymer coatings. C 1s peak component (*E*_b_ = 285.0 eV) served as reference for charge correction. Photoelectrons emitted orthogonal to the surface of the polymer coatings were analysed using energies of 20 eV for the region spectra and 160 eV for survey spectra. Data was collected at a step of 0.1 and 1 eV for the region and survey spectra, respectively. Linear background removal, quantification of elemental composition, deconvolution of peaks and fitting of the synthetic Gaussian-Lorentzian components to the peaks was performed using the instrument ESCape software (Kratos Analytical Ltd., Manchester, UK). The surface morphology was characterised using atomic force microscopy (AFM, NT-MDT NTEGRA Prima, Moscow, Russia) in a tapping mode.

### 3.2. Cells, Growth Conditions, Attachment and Viability

In this study, methicillin- and gentamicin-susceptible Gram-positive, round-shaped *Staphylococcus aureus* CIP 65.8^T^ bacteria was obtained from the Culture Collection of the Institute Pasteur (CIP, Paris, France), and Gram-negative *Pseudomonas aeruginosa* ATCC 9721 bacteria was acquired from the American Type Culture Collection (ATCC, Manassas, VA, USA). A 20% glycerol nutrient broth (Oxoid, Basingstoke, Hampshire, UK) was used to prepare bacterial stocks, which were then stored at −80 °C. Before every experiment, fresh bacterial suspensions of each of the strains in phosphate buffered saline (PBS) (10 mM, pH 7.4) were prepared. For *S. aureus*, the infective dose of 10^5^ cells per mL was used, whereas for *P. aeruginosa*, the infective dose of 10^3^ cells per mL was employed. Substrata were sterilized by first rinsing them with 70% ethanol and then exposing them to UV radiation for 30 min on each side. The stability of the polymer under different UV radiation conditions and when in contact with solvents has been previously reported [24,30,39]. Where samples were treated with plasma, decontamination took place prior to the plasma treatment step. An aliquot of 1 mL of suspension was then placed in each well of 24 well plate, and polymer-coated substrata were immersed into bacterial suspensions and allowed to incubate for 24 h at 22 °C in dark conditions without shaking. All experiments were conducted with at least three independent technical replicates.

The efficacy of polymer coatings in preventing settlement and colonization by bacterial cells was assessed by contact inoculation of pre-infected samples with culture plates and by liquid cultures. Sterile samples that were not exposed to bacterial cultures were used as controls. Upon retrieving samples from the 24 well culture plates, each sample was gently rinsed with copious amounts of PBS to remove unattached cells, and then allowed to come into direct contact with the surface of the sterile nutrient agar for 30 s, at which point the samples were removed. The agar plates were then transferred to an incubator, and allowed to incubate for 24 h at 37 °C and 5% CO_2_. The surfaces of the samples were additionally scraped with a sterile spatula, with the spatula then immersed into 5 mL culture media to transfer the scraped material. The liquid cultures were allowed to incubate for 24 h and 48 h at 37 °C and 5% CO_2_. Plates and liquid cultures without microbial growth after 24 h of incubation were assessed to be sterile. High-resolution electron micrographs of cells on surfaces were obtained using JSM 7400 SEM (JEOL, Tokyo, Japan) operated at 5 kV of polymer films.

### 3.3. Plasma Treatment of Surfaces and Growth Culture Medium

High-frequency (1.7 MHz, 2–6 kV) atmospheric pressure argon plasma jet was used for the treatment of both surfaces and of the growth culture medium. Even though plasmas generated using gas mixtures rich in oxygen, such as O_2_ gas and air, are significantly more effective in generating large quantities of biologically active RONS when compared to Ar plasmas, the latter are considered to be more stable and hence are frequently used in biomedical applications. Briefly, plasma is generated in the gap between the central electrode and ring electrode with argon flown through the cylinder at the rate of 3.0–5.0 standard liters per minute (SLM). For the treatment of polymer coatings, substrata were kept approximately 5 mm away from the nozzle so that the end of the plasma plume was just touching the surface. Samples were used immediately following the treatment. For plasma treatment of the culture medium, aliquots of 1 mL pure water were placed into a 24 well plate, and the plasma plume was allowed to touch the liquid. Immediately after plasma treatment, water containing bioactive species was added to the culture medium and used for the cell culture.

### 3.4. Eukaryotic Cell Line, Growth Conditions, Attachment and Viability

A mouse osteoblast precursor cell line, MC3T3-E1, and a mouse fibroblast like connective tissue line, L929, were used to assess the biocompatibility of samples after plasma exposure. Prior to seeding on the surfaces of substrata, MC3T3 cells were cultured in the α-MEM cell culture medium with the addition of 10% foetal bovine serum (FBS), and 1% penicillin-streptomycin (all from Invitrogen, Australia) at 37 °C and 5% CO_2_. Fibroblast cells were cultured in Dulbecco’s Modified Eagle Medium (DMEM) cell culture medium with the addition of 10% FBS, 1% L-glutamine, and 1% antibiotic/antimycotic penicillin-streptomycin (all from Invitrogen, Australia). Substrata were sterilized by first rinsing them with 70% ethanol and then exposing them to UV radiation for 30 min on each side. After sterilization and drying, substrata were placed into 24-well plates and incubated for 60 min with 20 µL of serum-free medium. 40 µL aliquots of either MC3T3 and L929 cells suspended in culture medium were transferred into each well using a top seeding static method. The plates were incubated for 2  h at 37  °C to facilitate initial attachment of cells to the surfaces. A culture medium (1 mL) was then gently added to each well-in some experiments, and a plasma activated culture medium was used in place of a standard culture medium to test the effect of plasma generated active species on the attachment and proliferation of cells, with standard culture medium as a control. The plates were then transferred to the incubator, where cells were kept at 5% CO_2_ and 37 °C in a humidified incubator. The medium was changed every day, being replaced with a standard medium in the control groups, and freshly prepared plasma activated medium in the experimental groups. Phase contrast imaging and SEM were used to visualise attachment patterns and cell morphology. Prior to imagining, cells were fixed with glutaraldehyde (3%) in cacodylate buffer (0.1 M) (Sigma) at 4  °C overnight. Once fixed, substrata were rinsed in sodium cacodylate buffer (0.1 M), osmium tetroxide (1%), deionized water, and dehydrated through a graded series of EtOH, then incubated in hexamethyldisilazane (HMDS) twice, for 30  min. All reagents were sourced from ProSciTech, Australia. The samples were allowed to fully air dry, then a thin gold coating was deposited on their surface via sputtering, and samples were scanned using SEM.

## 4. Conclusions

In summary, these results suggest that a decontamination-related surface treatment or exposure to plasma-activated media may in some instances have a measurable effect on the ability of biomaterial surfaces to retard microbial attachment or sustain eukaryotic cell growth. The exact impact of the treatment on the biological activity of polymer films depends on the combination of physical characteristics of these materials, in particular their surface and bulk chemistry, the nature of the treatment (e.g., the length of plasma exposure), the characteristics of the cell (e.g., strains, and whether the inoculum was derived from a mature biofilm or from broth culture), and the chemistry of the growth medium (in this study, the presence of plasma-generated biochemically-active RONS species). On one hand, this may present a challenge for the application of bio-active coatings in areas where in situ plasma decontamination is likely, e.g., in dentistry or over surgical sutures. On the other hand, there is an opportunity to develop polymer systems that exploit and benefit from being exposed to such plasma decontamination treatment.

## Figures and Tables

**Table 1:** Roughness characteristics, atomic fraction and chemical states of elements present in top surface layer of polymer coatings deposited on the surface of PET substrata.

	PET	10 W	15 W	20 W	25 W	30 W
1 µm × 1 µm						
*R*_q_, nm	1.07	3.19	5.98	2.20	1.73	8.97
*R*_max_, nm	4.31	22.49	27.12	10.87	7.64	33.75
*R*_min_, nm	−3.14	−7.49	−18.19	−6.90	−6.96	−28.25
*ADv*, nm	0.83	2.37	4.80	1.76	1.36	7.20
*R*_sk_	0.39	1.38	0.28	0.39	0.26	0.06
*R*_ku_	0.47	3.33	0.04	0.13	0.32	-0.15
10 µm × 10 µm						
*R*_q_, nm	2.08	4.72	5.19	4.07	3.21	9.18
*R*_max_, nm	39.73	56.82	54.84	38.02	36.58	107.03
*R*_min_, nm	−6.41	−14.28	−20.28	−11.85	−10.58	−31.72
*ADv*, nm	1.54	3.29	3.93	2.94	2.35	6.78
*R*_sk_	1.80	2.12	0.93	1.57	1.34	1.29
*R*_ku_	14.20	10.10	2.77	5.73	5.14	7.12
Atomic fraction, %						
O 1s (530 eV)		25.1	26.0	22.9	14.0	21.4
C 1s (285 eV)		73.0	74.0	77.1	86.1	78.7
N 1s (397 eV)		1.9				
O/C ratio		0.34	0.35	0.30	0.16	0.27
Water contact angle, °		62.1 ± 2.1	66.6 ± 1.9	70.3 ± 1.5	74.2 ± 1.8	77.1 ± 0.8

**Table 2:** Roughness characteristics, atomic fraction and chemical states of elements present in the top surface layer of polymer coatings deposited on the surface of PET substrata at 15 W before and after 60 s of plasma treatment.

	Before		After	
Surface roughness				
	1 µm × 1 µm	10 µm × 10 µm	1 µm × 1 µm	10 µm × 10 µm
*R*_q_, nm	5.98	5.19	7.33	5.45
*R*_max_, nm	27.12	54.84	29.76	34.29
*R*_min_, nm	−18.19	−20.28	−22.00	−18.71
*ADv*, nm	4.80	3.93	5.93	4.26
*R*_sk_	0.28	0.93	0.38	0.63
*R*_ku_	0.04	2.77	−0.09	0.91
Atomic fraction, %				
O 1s (530 eV)	26.0		29.6	
C 1s (285 eV)	74.0		70.4	

## Data Availability

The data presented in this study are available on request from the corresponding authors.

## Sample Availability

Samples are available from the authors.

## References

[1] Slepička P., Rimpelová S., Slepičková Kasálková N., Fajstavr D., Sajdl P., Kolská Z., Švorčík V. (2021). Antibacterial properties of plasma-activated perfluorinated substrates with silver nanoclusters deposition. Nanomaterials.

[2] van Hengel I.A.J., Tierolf M.W.A.M., Fratila-Apachitei L.E., Apachitei I., Zadpoor A.A. (2021). Antibacterial titanium implants biofunctionalized by plasma electrolytic oxidation with silver, zinc, and copper: A systematic review. Int. J. Mol. Sci..

[3] Griesser S.S., Jasieniak M., Vasilev K., Griesser H.J. (2021). Antimicrobial peptides grafted onto a plasma polymer interlayer platform: Performance upon extended bacterial challenge. Coatings.

[4] Lee M.-J., Kwon J.-S., Jiang H.B., Choi E.H., Park G., Kim K.-M. (2019). The antibacterial effect of non-thermal atmospheric pressure plasma treatment of titanium surfaces according to the bacterial wall structure. Sci. Rep..

[5] Benčina M., Resnik M., Starič P., Junkar I. (2021). Use of plasma technologies for antibacterial surface properties of metals. Molecules.

[6] Sardella E., Palumbo F., Camporeale G., Favia P. (2016). Non-equilibrium plasma processing for the preparation of antibacterial surfaces. Materials.

[7] Vasilev K., Griesser S.S., Griesser H.J. (2011). Antibacterial surfaces and coatings produced by plasma techniques. Plasma Process. Polym..

[8] Irfan M., Polonskyi O., Hinz A., Mollea C., Bosco F., Strunskus T., Balagna C., Perero S., Faupel F., Ferraris M. (2019). Antibacterial, highly hydrophobic and semi transparent Ag/plasma polymer nanocomposite coating on cotton fabric obtained by plasma based co-deposition. Cellulose.

[9] Akhavan B., Bakhshandeh S., Najafi-Ashtiani H., Fluit A.C., Boel E., Vogely C., van der Wal B.C.H., Zadpoor A.A., Weinans H., Hennink W.E. (2018). Direct covalent attachment of silver nanoparticles on radical-rich plasma polymer films for antibacterial applications. J. Mater. Chem. B.

[10] Dimitrakellis P., Ellinas K., Kaprou G.D., Mastellos D.C., Tserepi A., Gogolides E. (2021). Bactericidal action of smooth and plasma micro-nanotextured polymeric surfaces with varying wettability, enhanced by incorporation of a biocidal agent. Macromol. Mater. Eng..

[11] Cavallaro A.A., Macgregor-Ramiasa M.N., Vasilev K. (2016). Antibiofouling properties of plasma-deposited oxazoline-based thin films. ACS Appl. Mater. Interfaces.

[12] Bazaka O., Bazaka K., Truong V.K., Levchenko I., Jacob M.V., Estrin Y., Lapovok R., Chichkov B., Fadeeva E., Kingshott P. (2020). Effect of titanium surface topography on plasma deposition of antibacterial polymer coatings. Appl. Surf. Sci..

[13] Kumar A., Al-Jumaili A., Prasad K., Bazaka K., Mulvey P., Warner J., Jacob M.V. (2020). Pulse plasma deposition of terpinen-4-ol: An insight into polymerization mechanism and enhanced antibacterial response of developed thin films. Plasma Chem. Plasma Process..

[14] Lu X., Feng X., Werber J.R., Chu C., Zucker I., Kim J.-H., Osuji C.O., Elimelech M. (2017). Enhanced antibacterial activity through the controlled alignment of graphene oxide nanosheets. Proc. Nat. Acad. Sci. USA.

[15] Prasad K., Bandara C.D., Kumar S., Singh G.P., Brockhoff B., Bazaka K., Ostrikov K.K. (2017). Effect of precursor on antifouling efficacy of vertically-oriented graphene nanosheets. Nanomaterials.

[16] Yick S., Mai-Prochnow A., Levchenko I., Fang J., Bull M.K., Bradbury M., Murphy A.B., Ostrikov K. (2015). The effects of plasma treatment on bacterial biofilm formation on vertically-aligned carbon nanotube arrays. RSC Adv..

[17] Mai-Prochnow A., Clauson M., Hong J., Murphy A.B. (2016). Gram positive and Gram negative bacteria differ in their sensitivity to cold plasma. Sci. Rep..

[18] Mitra S., Veerana M., Choi E.-H., Park G. (2021). Effects of pre-treatment using plasma on the antibacterial activity of mushroom surfaces. Foods.

[19] Grant D.S., Ahmed J., Whittle J.D., Michelmore A., Vasilev K., Bazaka K., Jacob M.V. (2021). Comparative study of natural terpenoid precursors in reactive plasmas for thin film deposition. Molecules.

[20] Zhou R., Zhou R., Prasad K., Fang Z., Speight R., Bazaka K., Ostrikov K. (2018). Cold atmospheric plasma activated water as a prospective disinfectant: The crucial role of peroxynitrite. Green Chem..

[21] Bazaka K., Jacob M.V., Bowden B.F. (2011). Optical and chemical properties of polyterpenol thin films deposited via plasma-enhanced chemical vapor deposition. J. Mater. Res..

[22] Bazaka K., Jacob M.V., Truong V.K., Wang F., Pushpamali W.A.A., Wang J.Y., Ellis A.V., Berndt C.C., Crawford R.J., Ivanova E.P. (2010). Plasma-enhanced synthesis of bioactive polymeric coatings from monoterpene alcohols: A combined experimental and theoretical study. Biomacromolecules.

[23] Bazaka K., Jacob M.V., Truong V.K., Crawford R.J., Ivanova E.P. (2011). The effect of polyterpenol thin film surfaces on bacterial viability and adhesion. Polymers.

[24] Bazaka K., Jacob M.V. (2010). Post-deposition ageing reactions of plasma derived polyterpenol thin films. Polym. Degrad. Stab..

[25] Bazaka K., Ketheesan N., Jacob M.V. (2014). Polymer encapsulation of magnesium to control biodegradability and biocompatibility. J. Nanosci. Nanotechnol..

[26] Kumar A., Al-Jumaili A., Bazaka K., Mulvey P., Warner J., Jacob M.V. (2020). In-situ surface modification of terpinen-4-ol plasma polymers for increased antibacterial activity. Materials.

[27] Kumar A., Mills S., Bazaka K., Bajema N., Atkinson I., Jacob M.V. (2018). Biodegradable optically transparent terpinen-4-ol thin films for marine antifouling applications. Surf. Coat. Technol..

[28] Jiang H., Grant J.T., Enlow J., Su W., Bunning T.J. (2009). Surface oxygen in plasma polymerized films. J. Mater. Chem..

[29] Kostov K.G., Nishime T.M.C., Castro A.H.R., Toth A., Hein L.R.O. (2014). Surface modification of polymeric materials by cold atmospheric plasma jet. Appl. Surf. Sci..

[30] Bazaka K., Ahmad J., Oelgemöller M., Uddin A., Jacob M.V. (2017). Photostability of plasma polymerized γ-terpinene thin films for encapsulation of OPV. Sci. Rep..

[31] Bazaka K., Bazaka O., Levchenko I., Xu S., Ivanova E.P., Keidar M., Ostrikov K. (2017). Plasma-potentiated small molecules—possible alternative to antibiotics?. Nano Futures.

[32] Zhou R., Zhou R., Zhuang J., Zong Z., Zhang X., Liu D., Bazaka K., Ostrikov K. (2016). Interaction of atmospheric-pressure air microplasmas with amino acids as fundamental processes in aqueous solution. PLoS ONE.

[33] Zhou R., Zhou R., Wang P., Luan B., Zhang X., Fang Z., Xian Y., Lu X., Ostrikov K.K., Bazaka K. (2019). Microplasma bubbles: Reactive vehicles for biofilm dispersal. ACS Appl. Mater. Interfaces.

[34] Dai X., Bazaka K., Richard D.J., Thompson E.W., Ostrikov K. (2018). The emerging role of gas plasma in oncotherapy. Trends Biotechnol..

[35] Kaushik N.K., Chimire B., Li Y., Adhikari M., Veerana M., Kaushik N., Jha N., Adhikari B., Lee S.-J., Masur K. (2019). Biological and medical applications of plasma-activated media, water and solutions. Biol. Chem..

[36] Schuster D.I., Baran P.S., Hatch R.K., Khan A.U., Wilson S.R. (1998). The role of singlet oxygen in the photochemical formation of C60O. Chem. Commun..

[37] Tominami K., Kanetaka H., Sasaki S., Mokudai T., Kaneko T., Niwano Y. (2017). Cold atmospheric plasma enhances osteoblast differentiation. PLoS ONE.

[38] Bremmell K.E., Kingshott P., Ademovic Z., Winther-Jensen B., Griesser H.J. (2006). Colloid probe AFM investigation of interactions between fibrinogen and PEG-like plasma polymer surfaces. Langmuir.

[39] Bazaka K., Jacob M.V. (2012). Solubility and surface interactions of RF plasma polymerized polyterpenol thin films. Mater. Express.

